# Combination Therapy with Fluoxetine and the Nucleoside Analog GS-441524 Exerts Synergistic Antiviral Effects against Different SARS-CoV-2 Variants In Vitro

**DOI:** 10.3390/pharmaceutics13091400

**Published:** 2021-09-03

**Authors:** Linda Brunotte, Shuyu Zheng, Angeles Mecate-Zambrano, Jing Tang, Stephan Ludwig, Ursula Rescher, Sebastian Schloer

**Affiliations:** 1 Institute of Virology, Center for Molecular Biology of Inflammation, and “Cells in Motion” Interfaculty Centre, University of Muenster, Von-Esmarch-Str. 56, D-48149 Muenster, Germany; brunotte@uni-muenster.de (L.B.); a_meca01@uni-muenster.de (A.M.-Z.); ludwigs@uni-muenster.de (S.L.); 2 Research Program in Systems Oncology, Faculty of Medicine, University of Helsinki, Haartmaninkatu 8, 00029 Helsinki, Finland; shuyu.zheng@helsinki.fi (S.Z.); jing.tang@helsinki.fi (J.T.); 3 Institut-Associated Research Group Regulatory Mechanisms of Inflammation, Institute of Medical Biochemistry, Center for Molecular Biology of Inflammation, and “Cells in Motion” Interfaculty Centre, University of Muenster, Von-Esmarch-Str. 56, D-48149 Muenster, Germany; rescher@uni-muenster.de

**Keywords:** combination therapy, SARS-CoV-2, nucleoside GS-441524, fluoxetine, synergy

## Abstract

The ongoing SARS-CoV-2 pandemic requires efficient and safe antiviral treatment strategies. Drug repurposing represents a fast and low-cost approach to the development of new medical treatment options. The direct antiviral agent remdesivir has been reported to exert antiviral activity against SARS-CoV-2. Whereas remdesivir only has a very short half-life time and a bioactivation, which relies on pro-drug activating enzymes, its plasma metabolite GS-441524 can be activated through various kinases including the adenosine kinase (ADK) that is moderately expressed in all tissues. The pharmacokinetics of GS-441524 argue for a suitable antiviral drug that can be given to patients with COVID-19. Here, we analyzed the antiviral property of a combined treatment with the remdesivir metabolite GS-441524 and the antidepressant fluoxetine in a polarized Calu-3 cell culture model against SARS-CoV-2. The combined treatment with GS-441524 and fluoxetine were well-tolerated and displayed synergistic antiviral effects against three circulating SARS-CoV-2 variants in vitro in the commonly used reference models for drug interaction. Thus, combinatory treatment with the virus-targeting GS-441524 and the host-directed drug fluoxetine might offer a suitable therapeutic treatment option for SARS-CoV-2 infections.

## 1. Introduction

The Coronavirus Disease 2019 (COVID-19) caused by the Severe Acute Respiratory Syndrome Related Coronavirus 2 (SARS-CoV-2) has resulted in over 2 million deaths within one year and demonstrates the risk of newly emerged pathogens [1,2]. 

In contrast to other human circulating coronaviruses, SARS-CoV-2 leads to a severe disease with multiple organ failures, especially in elderly patients and those with chronic medical conditions [3,4,5]. Although vaccines are available, their production, distribution and vaccine hesitancy are critical limiting factors in healthcare. Thus, additional therapeutic strategies to combat the SARS-CoV-2 infection are needed. However, the development and production of new antiviral drugs is a time-consuming process that can be accelerated by the repurposing of already clinically licensed drugs [6,7].

One of the repurposed FDA-approved drugs that has received considerable attention as an antiviral agent against SARS-CoV-2 is remdesivir, a nucleotide monophosphate analogue of adenosine monophosphate (AMP) that interferes with the viral RNA-dependent RNA polymerase [8,9]. Remdesivir was originally developed by Gilead for the treatment of Ebola [10], and is shown to have strong therapeutic efficacy in in vivo models of coronaviruses (MERS-CoV, SARS-CoV, SARS-CoV-2) in mice and primates [11,12,13]. However, it has a very limited half-life time in the plasma of patients [14,15,16]. Remdesivir is converted into its predominant serum metabolite GS-441524, which maintains the antiviral properties [12,15,16,17,18]. A study conducted in rhesus macaques infected with SARS-CoV-2 treated with remdesivir revealed 1000-fold higher GS-441524 serum levels than those of remdesivir [16]. The benefit of GS-441524 over remdesivir is the lower molecular weight and hydrophilicity, which makes it easier to produce an aerosolized formulation for inhalable therapeutic treatment. An inhalable formulation would allow a high concentration of the drug in lung cells and minimized systemic toxicity [17]. Hence, GS-441524 has a higher potential to be used for antiviral treatments of respiratory pathogens like SARS-CoV-2. 

While the majority of antiviral drugs such as remdesivir or GS-441524 are directly targeting viral proteins and are quite efficient to eliminate the pathogen, they pose the risk of emerging viral resistance [19,20,21]. Thus, combination therapies that include virus- and host-directed drugs are considered to cause less resistance. We recently reported the importance of the endosomal lipid balance for the entry process of enveloped viruses like SARS-CoV-2. The clinically licensed antidepressant fluoxetine, a drug belonging to the class of functional inhibitors of acid sphingomyelinase (FIASMA), blocks the sphingomyelin converting acid sphingomyelinase (ASMase) within the late endosomal/lysosomal (LEL) compartments [22]. The inhibitory effects of fluoxetine relies on its ability to interfere with the endosomal lipid balance, preventing the entry of SARS-CoV-2 [23]. 

Here, we evaluated the antiviral potential of GS-441524 in a polarized Calu-3 cell culture model when administered alone or in combination with the host-directed drug fluoxetine. The drug combination of fluoxetine and GS-441524 showed stronger antiviral activities against three different SARS-CoV-2 variants compared to the monotherapies. Notably, both drugs act synergistic, as calculated with the commonly used reference models for drug interaction studies. 

## 2. Materials and Methods

### 2.1. Cells and Compounds

The human bronchial epithelial cell line Calu-3 and the Vero E6 cells derived from the kidney of an African green monkey were cultivated in Dulbecco’s modified Eagle’s medium (DMEM, Sigma-Aldrich, Darmstadt, Germany) with a 10% standardized fetal bovine serum (FBS Advance; Capricorn, Ebsdorfergrund, Germany), 2 mM L-glutamine, 100 U/mL penicillin, 0.1 mg/mL streptomycin, and 1% non-essential amino acids (Merck, Darmstadt, Germany) in a humidified incubator at 5% CO_2_ and 37 °C. Calu-3 monolayers were polarized and cultured as described [24]. Fluoxetine (5 mM, Sigma-Aldrich, Darmstadt, Germany) and GS-441524 (100 mM, Biomol, Hamburg, Germany) were solubilized in DMSO. 

### 2.2. Cytotoxicity Assay

Calu-3 cells were cultured at the indicated concentrations with either the solvent DMSO, GS-441524, fluoxetine or with the combinations of fluoxetine/GS-441524 for 48 h. To estimate cytotoxic effects, a staurosporine solution (1 μM) was used as a positive control. The cell viability was evaluated by adding MTT 3-(4,5-dimethylthiazol-2-yl)-2,5-diphenyltetrazolium bromide (Sigma-Aldrich, Darmstadt, Germany) to the cells for 4 h and OD_562_ measurements according to the manufacturer’s protocols (Sigma-Aldrich, Darmstadt, Germany).

### 2.3. Virus Infection and Drug Treatment

The Muenster SARS-CoV-2 isolate hCoV-19/Germany/FI1103201/2020 (EPI-ISL_463008, mutation D614G in spike protein), and the two newly emerged variants B1.1.7 UK VOC (alpha) and B1.351 SA VOV (beta) were amplified on Vero E6 cells (passage 1) and used for the infection assays. Polarized Calu-3 cells were washed once with PBS and inoculated with the virus diluted in infection-PBS (containing 0.2% BSA, 1% CaCl_2_, 1% MgCl_2_, 100 U/mL penicillin and 0.1 mg/mL streptomycin) at a multiplicity of infection (MOI) of 0.1 at 37 °C for 1 h. Following infection, cells were washed with PBS and cultured in infection-DMEM (serum-free DMEM containing 0.2% BSA, 1 mM MgCl_2_, 0.9 mM CaCl_2_, 100 U/mL penicillin, and 0.1 mg/mL streptomycin) at 5% CO_2_ and 37 °C. Calu-3 cells were then treated with the solvent DMSO or the indicated GS-441524 or fluoxetine concentration at 2 h post-infection (hpi) for the entire 48 h infection period. Afterwards, the apical culture supernatants were collected and immediately frozen at −80 °C to determine the number of infectious particles.

### 2.4. Plaque Assay 

The number of infectious particles in the supernatant of treated cells were governed via a standard plaque assay. Briefly, monolayers of Vero E6 cells cultured in six-well dishes were washed with PBS and infected with serial dilutions of the respective supernatants in infection-PBS for 1 h at 37 °C. Subsequently, the inoculum was replaced with 2x MEM (MEM containing 0.2% BSA, 2 mM L-glutamine 1 M HEPES, pH 7.2, 7.5% NaHCO_3_, 100 U/mL penicillin, 0.1 mg/mL streptomycin, and 0.4% Oxoid agar) and incubated at 37 °C for 72 h. A neutral red staining was performed to visualize virus plaques, and virus titers were calculated and expressed as plaque-forming units (PFU) per mL.

### 2.5. Data and Statistical Analysis

The required sample sizes (to detect a > 90% reduction in virus titers at a power > 0.8) were determined by using the a priori power analysis G*Power 3.1 (Faul et al., 2007). Data were analyzed using the software GraphPad Prism version 8.00 (GraphPad). 

To define dose–response curves, virus titers were normalized to the percentages of titers detected in cells treated with the solvent DMSO (control), and drug concentrations were log-transformed. EC values were calculated from the sigmoidal curve fits using a four-parameter logistic (4PL) model. The combinatory effects of the drug pair fluoxetine/ GS-441524 were analyzed by using SynergyFinder, an open-source, free, stand-alone web application for the analysis of drug combination data [25]. The synergy was evaluated based on the Zero Interaction Potency (ZIP), Bliss independence, and highest single agent (HSA) reference models. Additionally, we analyzed the overall drug combination sensitivity score (CSS) by using the CSS method [26]. For statistical analysis of cytotoxicity assays, values were normalized to the percentages of toxicity detected in the control cells (cells treated with the solvent DMSO); significant differences were evaluated using a one-way ANOVA followed by Dunnett’s multiple comparison test. ** *p* < 0.01, *** *p* < 0.001, **** *p* ≤ 0.0001.

## 3. Results

**Figure 1:**
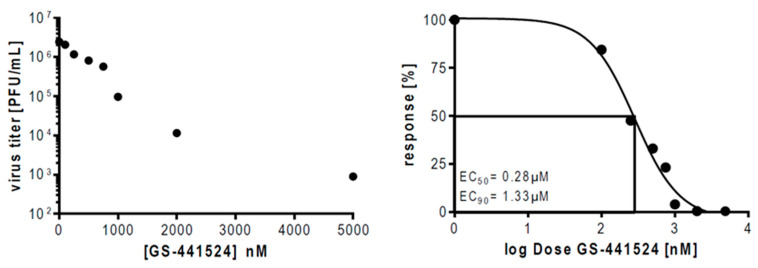
Analysis of GS-441524-mediated reduction of infectious SARS-CoV-2 particle production. Polarized Calu-3 cells were infected with 0.1 MOI of SARS-CoV-2 (hCoV-19/Germany/FI1103201/2020) for 48 h. At 2 hpi, cells were treated with GS-441524 at the indicated concentrations. Data were expressed as mean infectious viral titers ± SEM or as mean percent inhibition ± SEM of SARS-CoV-2 replication (control cells that were treated with the solvent DMSO were set to 100%), *n* = 5. LogEC_50_ and LogEC_90_ values were determined by fitting a four-parameter non-linear regression model.

We have recently reported that the clinically used antidepressant fluoxetine in combination with the viral RNA-dependent RNA polymerase inhibitor remdesivir exhibits synergistic antiviral effects against the SARS-CoV-2 infection in vitro [27]. A major drawback for the in vivo use of the prodrug remdesivir is the very short plasma half-life time of approximately 20 min [17]. Remdesivir is converted into its main plasma metabolite GS-441524 when administered to patients [14,17]. Thus, we wanted to assess the antiviral potential of GS-441524 in a polarized Calu-3 cell culture model. We infected Calu-3 cells with the isolate hCoV-19/Germany/FI1103201/2020 at MOI 0.1 for 48 h and quantified the production of infectious SARS-CoV-2 particles by a plaque assay. Control Calu-3 cells that were treated with the solvent DMSO yielded viral titers up to 2 × 10^6^ PFU, whereas treatment with the nucleoside GS-441524 2hpi significantly inhibited the production of the circulating SARS-CoV-2 variant in a dose-depended manner (Figure 1). Fitting of the experimental dose–response values to a nonlinear four-parameter logistic model resulted in a half-maximal inhibitory (EC_50_) and 90% inhibitory concentrations (EC_90_) of 0.28 µM and 1.33 µM, respectively, for the Muenster Isolate (Figure 1). Validation of Calu-3 cell viability after administration of GS-441524 via an MTT assay revealed that only a very high concentration of GS-441524 resulted in detectable cytotoxicity, whereas all concentrations further used in the pharmacological interaction studies had no influence on the cell viability (Figure S1a, Supplementary Material). The calculated 50% cytotoxic concentration (CC_50_) of the remdesivir metabolite is 47.66 µM with a selectivity index (SI) of 170.21, which emphasizes a safe antiviral treatment window.

**Figure 2:**
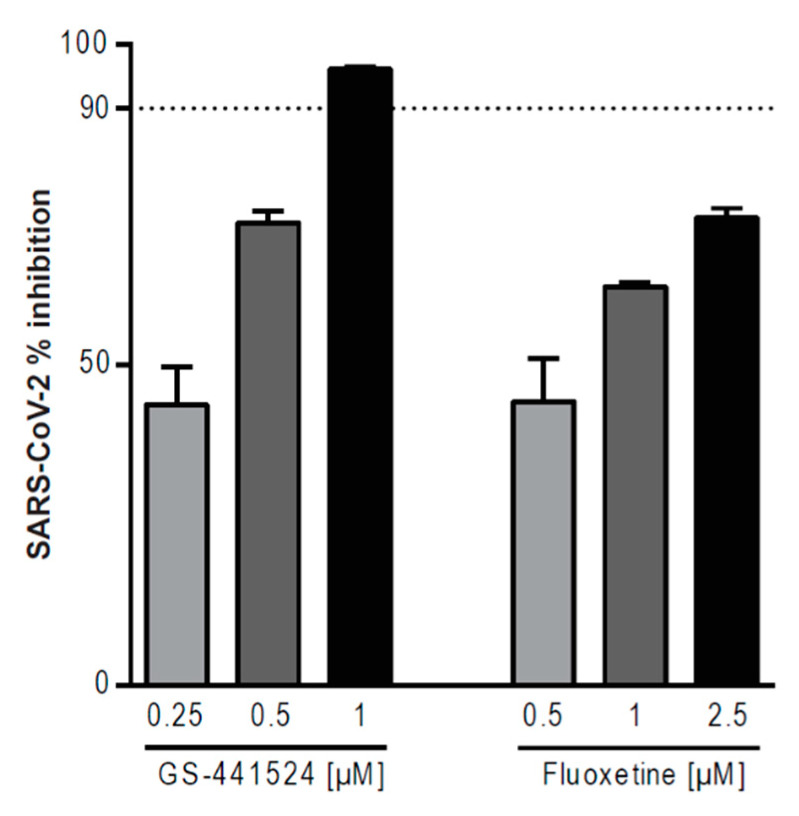
Antiviral activities of a single treatment against SARS-CoV-2. Polarized Calu-3 cells were infected with SARS-CoV-2 and treated with the indicated GS-441524 or fluoxetine concentrations for 48 h. Bars represent mean percent inhibition ± SEM of infectious virus production, with mean virus titer produced in control cells (treated with the solvent DMSO) set to 100%; *n* = 5. Dotted line, 90% reduction in viral titer.

We next addressed whether a combinatory treatment with the drug pair fluoxetine-GS-441524 had a synergistic interaction to limit the SARS-CoV-2 infection. For studying the antiviral properties of the drug combinations, we used, for both drugs, concentrations that were previously reported to have an individual antiviral activity below 90%, whereas their combination was able to achieve a more than 90% reduction in viral titers [27]. The highest single dose of GS-441524 (1000 nM) was able to achieve 95% inhibition on viral titers, whereas treatment with the highest single dose of fluoxetine reduced viral titers up to 75% (Figure 2).

**Figure 3:**
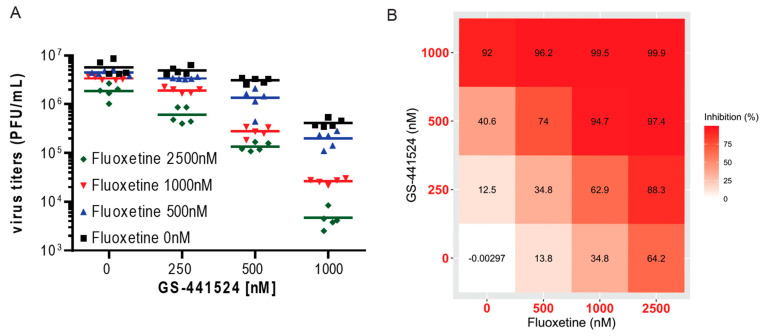
Antiviral activities of combination treatment with fluoxetine and GS-441524. Polarized Calu-3 cells were infected with SARS-CoV-2 and treated with the indicated drug combinations for 48 h. (**A**) Data were expressed as plaque-forming units (PFU) per mL detected in a single experimental sample, lines indicate means; *n* = 5/treatment or as (**B**) percent pharmacological inhibition of infectious virus production for the drug pair fluoxetine and GS-441524 (with mean virus titer produced in control cells (treated with the solvent DMSO) set to 100%).

Next, we determined the number of infectious virus particles in Calu-3 cells that weretreated with a combination of both drugs. On the basis of our recent publications [27] on the antiviral potential of fluoxetine alone or in combination with remdesivir, we now analyzed the antiviral effects of a combined fluoxetine/GS-441524 treatment (Figure 3A,B). We observed a noticeable increase in the pharmacological inhibition of infectious virus production (>90%) when cells were treated with a concentration of 500 nM GS-441524 and 1000 nM fluoxetine or higher doses of the drug pair (Figure 3A,B), thus showing the great potential of a combination treatment of the remdesivir metabolite GS-441524 with fluoxetine. Additionally, we assessed the cytotoxic effects of the combinatory treatments via an MTT assay to exclude the potential synergistic toxicity of the drug pair. The MTT assay is based on the reduction in 3-(4,5-dimethylthiazol-2-yl)-2,5-diphenyltetrazolium bromide to formazan crystals by NAD(P)H-dependent oxidoreductase enzymes in metabolically active cells, this colorimetric assay measures the metabolic activity as an integrated indicator of changes in the cell viability, cytotoxicity, and proliferation. As the analysis of the combination treatments with fluoxetine and GS-441524 did not reveal any toxicities (Figure S1b), we continued to analyze the drug synergy without the subtraction of cytotoxicity.

**Figure 4:**
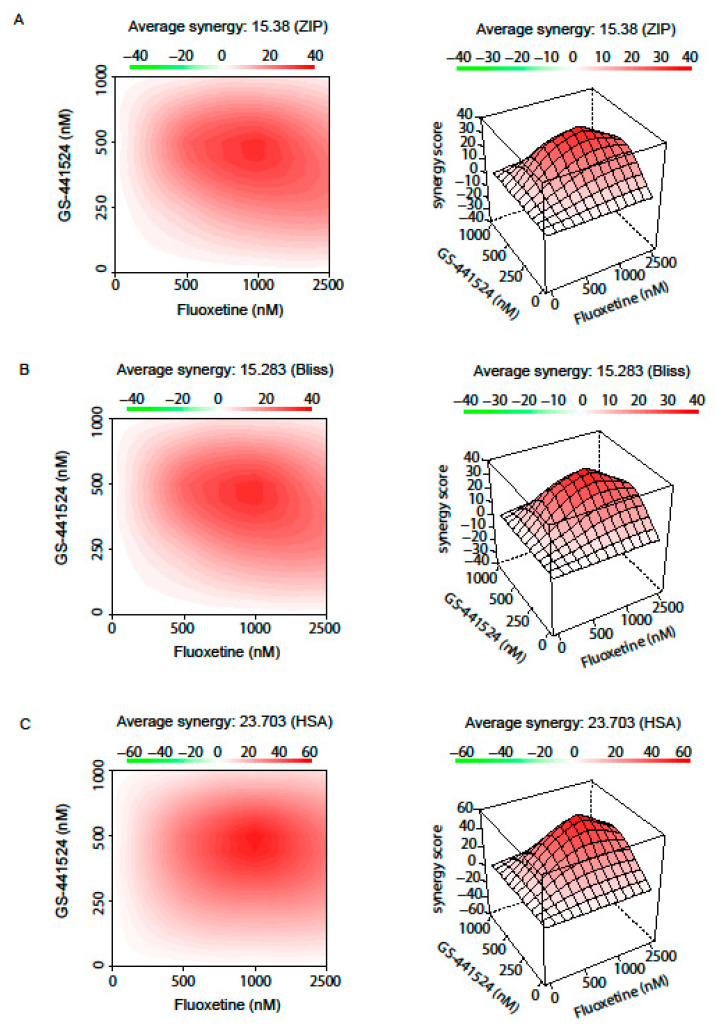
Pharmacological interaction profile of the drug pair GS-441524 and fluoxetine. Drug interactions were analyzed based on the three commonly used reference models: (**A**) Zero Interaction Potency (ZIP), (**B**) Bliss independence, and (**C**) highest single agent (HSA). While the HSA model assumes a synergistic drug combination that produce additional benefits on top of what the drugs can achieve alone, the Bliss independence model uses probabilistic theory to model the effects of individual drugs in a combination as independent yet competing events. Synergy calculations via the ZIP model includes the comparison of potency changes of the dose–response curves between individual drugs and their combinations. A color-coded interaction surface was used to illustrate the synergy scores of the responses, where high synergistic scores are colored in red. Synergy score calculations via the ZIP and Bliss independence model revealed a synergy of ~ 15, while the HSA model showed a higher synergy score of ~23.

Although drug synergy is not necessarily required for clinical benefits, synergy scoring remains an important parameter for the evaluation of drug combination therapies. Thus, we next evaluated the drug interaction profile of fluoxetine and GS-441524 by using three commonly used reference synergy models: ZIP, Bliss independence and highest single agent (HSA). Even though these different reference synergy models analyzed the drug interactions based on different basic interaction assumptions, they emphasized a synergistic action of GS-441524 and fluoxetine (Figure 4). The drug interaction relationships and landscape visualizations revealed in all models, a high synergy score when cells were treated with a combination of 500–1000 nM GS-441524 and ~1000 nM fluoxetine. The strong synergy of the combinatory treatment with both drugs led to an overall drug combination sensitivity score (CSS) of 92.42.

**Figure 5:**
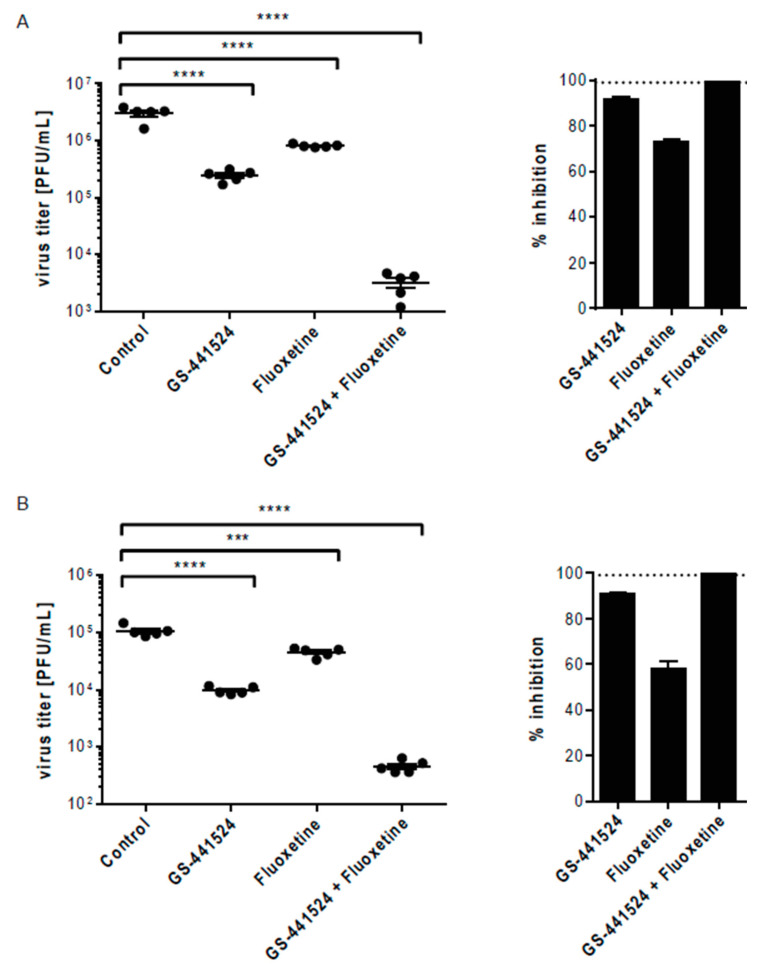
Antiviral effect of the combination therapy with GS-441524 and fluoxetine against two newly emerged SARS-CoV-2 variants. Polarized Calu-3 cells were infected with the (**A**) alpha or (**B**) beta variant of SARS-CoV-2 and treated with 2.5 µM fluoxetine, 1 µM GS-441524 or the combination of both drugs for 48 h. (**A**) Data were expressed as plaque-forming units (PFU) per mL detected in a single experimental sample, lines indicate means; *n* = 5/treatment or as (**B**) percent pharmacological inhibition of infectious virus production for the drug pair fluoxetine and GS-441524 (with mean virus titer produced in control cells (treated with the solvent DMSO) set to 100%, *n* = 5. Dotted line, 99% reduction in viral titer. One-way ANOVA followed by Dunnett’s multiple comparison test. *** *p* ≤ 0.001, **** *p* ≤ 0.0001.

We further assessed the antiviral capacity of the combination therapy with GS-441524 and fluoxetine against the SARS-CoV-2 alpha and beta variants of concern (VOC). Both strains have mutations in the spike protein’s receptor binding domain (for example, 501Y, a change from asparagine (N) to tyrosine (Y) in amino-acid position 501), which impair angiotensin-converting enzyme 2 (ACE2) binding specificity and lead to an increased transmissibility [28,29,30,31]. At least for the beta variant, changes in the spike protein’s receptor-binding domain enables a partial immune escape from neutralization induced by vaccination or previous virus infection and is therefore considered to be of concern [28]. Importantly, the combination of GS-441524 and fluoxetine potently reduced viral titers of both VOCs synergistically when compared to monotherapy (Figure 5). While the monotherapy reduced viral titers between 60 to 70% for fluoxetine or up to 90% when treated with GS-441524, the combination of both drugs resulted in a viral inhibition above 99% (Figure 5). Thus, the combination of the host-directed fluoxetine and the virus-targeting GS-441524 showed great antiviral potential against SARS-CoV-2 variants that have significant changes in the spike protein’s receptor-binding domain.

## 4. Discussion

Emerging zoonotic diseases such as the current SARS-CoV-2 pandemic are global threats to humans and the health care systems. SARS-CoV-2, which causes COVID-19, has already led to more than 2 million deaths within one year. Thus, vaccines and antivirals are urgently needed to decelerate the global spreading and community transmission of SARS-CoV-2. Antiviral therapy often includes a combination of several drugs, each targeting different steps in the virus life-cycle to circumvent the emergence of drug resistance. The benefit of antiviral combinations has been reported in a large number of studies [32,33,34,35]. The most significant and latest successes of antiviral combination therapy was achieved in the fight against HIV-1 or HCV, where drugs that interfere with the virus entry and replication were used [36,37,38]. While host-directed drugs mostly impair the viral replication without a complete eradication of the pathogen, antivirals that directly target viral proteins are much more efficient in eradicating viruses. However, a major concern of direct antiviral therapy is the risk to induce new resistant virus strains [39], an adaptive step that was already observed in the antiviral therapy against influenza or HIV [40,41]. The combination of antivirals with host-directed drugs makes it much more unlikely that a virus can overcome the antiviral barrier and emerge resistances. Thus, the combination of both is routinely explored for enhanced treatment success [42,43,44]. 

One critical step in the life cycle of enveloped viruses such as SARS-CoV-2 is the entry into the host cell. SARS-CoV-2, similar to other enveloped viruses, needs to overcome the host cell membrane for transferring the viral genome into the cytosol, a step that is limited by the fusion of viral and cellular membranes [23,45]. SARS-CoV-2 binds via its spike protein, a viral envelope protein, to the host cell receptor ACE2 [46,47,48]. Attachment of virus particles facilitate a priming of the spike protein via proteolytic cleavage, which is mediated by several host proteases and a prerequisite for membrane fusion. Cleavage by the cellular transmembrane protease serine 2 (TMPRSS2) triggers the fusion with the plasma membrane, whereas other endosome-residing proteases are required for the fusion of endocytosed SARS-CoV-2 particles with endosomes [23,45]. Thus, the endosomal compartment is a critical host/pathogen interface for SARS-CoV-2 [23]. The antiviral mode of action of fluoxetine is most likely based on its inhibitory effect on the endolysosome-residing enzyme sphingomyelin phosphodiesterase (“acid sphingomyelinase”, ASM). The blocking of ASM activity results in sphingomyelin accumulation, which negatively affects cholesterol release from the endolysosomal compartment, causing the favored antiviral barrier [22,23].

In our recent study [27], we showed that the combination of the host-directed drug fluoxetine and the viral RNA-dependent RNA-polymerase inhibitor remdesivir results in a synergistic antiviral effect on the production of infectious virus particles. Remdesivir was originally developed for the treatment of Ebola [18], but exerts antiviral activity against a number of other viruses, including Ebolavirus, Marburg virus, MERS-CoV and also SARS-CoV-2 [9,11,15,18,49]. Remdesivir was the first drug that received an FDA emergency use authorization for severe COVID-19 treatment. Since remdesivir has a very short half-life time in the plasma of patients (approximately 20 min) and, moreover, requires activation through pro-drug enzymes (such as carboxylesterases (CES1), cathepsin A (CTSA) and histidinetriad nucleotide binding proteins (HINT)) which are preferentially expressed in the liver [17,50,51,52,53], it is unsuitable for a lung-specific delivery and its clinical use remains controversial [17]. Structural similarity studies between the main remdesivir metabolite GS-441524 and human enzymes suggest that the bioactivation of GS-441524 relies on adenosine kinase (ADK) [17]. ADK is moderately expressed across all tissues and, thus, the administration of GS-441524 would be more eligible for systemic and lung-specific delivery. GS-441524 has been reported to potently inhibit SARS-CoV-2 replication in vitro and in a mouse model of SARS-CoV-2 infection and pathogenesis [11,12], implying this metabolite as a promising drug candidate for further evaluation. The favorable safety profile of GS-441524 (shown by the better SI values [10,54] and by animal models [55,56]) suggests an increased therapy window, which allows for a higher dosing of GS-441524 compared to remdesivir without causing adverse side effects. 

Our data are consistent with recent studies demonstrating that the monotherapy of the remdesivir metabolite GS-441524 elucidated similar EC_50_ and EC_90_ values similar to remdesivir in polarized Calu-3 cells (GS-441524; EC_50_ = 0.28 µM and EC_90_ = 1.33 µM; remdesivir: EC_50_ = 0.28 μM, EC_90_ = 2.48 μM, ref. [11]). 

We further evaluated the overall antiviral effect of the combination GS441524 and fluoxetine, which was larger than the expected sum of the independent drug effects, showing a synergistic effect against three circulating SARS-CoV-2 variants (Figure 4 and Figure 5). Treatment of GS-441524 in combination with fluoxetine indicates a comparable synergistic activity to the recent published combination of fluoxetine and remdesivir [27]. Both combination treatments lead to an average synergy score of ~15 (in the ZIP or Bliss independence reference model) or of ~23 in the HSA reference model with a high synergy score when cells were treated with a combination of 500–1000 nM GS-441524 or remdesivir and 1000–2500 nM fluoxetine [27]. Of note, no cytotoxic effects were observed when the cells were treated with the combination of both drugs. For successful monotherapy of the individual drugs, high drug doses are required, and a prolonged treatment is often associated with poor patient compliance. The synergistic action of fluoxetine and GS-441524 offers the administration of lower concentrations of the individual drugs, which can reduce potential side effects.

The transfer of in vitro data to the in vivo situation is critical in antiviral research. Thus, we compared the concentrations shown to be effective in our in vitro study with reachable plasma concentrations in patients when drugs were administered. The nucleoside analog GS-441524 can reach plasma concentrations up to 1000-fold higher than remdesivir (maximum plasma levels 3 mg/L directly after intravenous infusion and 80–170 µg/L after 1 h when given intravenously) [14], whereas orally administered fluoxetine (20 mg/day) has a high bioavailability with plasma levels of 350 µg/L after two weeks and up to 1055 µg/L for longer treatment periods in patients [57,58]. For both drugs, plasma concentrations are well within the ranges that equal effective drug concentrations in vitro.

Our results demonstrate a strain-independent potential therapeutic capacity of combined treatment with the direct antiviral acting nucleoside analog GS-441524 and the host-directed drug fluoxetine to combat the SARS-CoV-2 infection and limit deleterious COVID-19 outcomes. At least mutations occurring in the spike protein’s receptor binding domain had no influence on the antiviral efficacy of the combination or monotherapies with GS-441524 and/or fluoxetine (Figure 5) [28,29,30,31]. The eligibility of combining host-directed drugs with antivirals in SARS-CoV-2 therapy was recently confirmed in a double-blind, randomized, placebo-controlled trial where combination therapy with remdesivir and the host-directed Janus kinase inhibitor baricitinib was beneficial in the treatment of hospitalized COVID-19 patients [59,60].

However, combined medications pose the risk of drug–drug interactions which may lead to a reduced therapeutic benefit or even severe adverse effects. Thus, it is indispensable to survey the drug interactions and to carefully evaluate the appropriate treatment strategy against SARS-CoV-2. While clinical data from healthy donors showed that remdesivir and its metabolite GS-4412524 are metabolized through Cytochromes P450 (CYPs) enzymes (CYP2C8, CYP2D6, and CYP3A4), clinical studies that examined drug–drug interactions were not yet complete, although the mathematical prediction of DDI liability suggested that remdesivir and GS-441524 might elevate the levels of co-prescribed drugs that depend on these CYP enzymes [61,62,63]. However, the influence of remdesivir on CYP-enzyme dependent metabolism is suggested to be weak [61,63]. Thus, simultaneous administration with fluoxetine, another known inhibitor of CYPs (CYP2D6 and CYP2C9/10) should be carefully monitored [64,65,66]. As fluoxetine is also a serotonin-reuptake inhibitor (SRI), simultaneous administration with other SRIs should also be avoided (including amphetamines and other sympathomimetic appetite suppressants) [67,68]. For further information about possible drug–drug interaction, visit Drugs.com (accessed on 18 February 2021) [69]. Since fluoxetine can exert, in some patients, serious side effects, we do not recommended self-medication. The careful administration of drugs should exclusively rely on medical advice.

## Data Availability

The data presented in this study are available on request from the corresponding author. The data are not publicly available due to privacy or ethical restrictions.

## Supplementary Materials

The following are available online at https://www.mdpi.com/article/10.3390/pharmaceutics13091400/s1, Figure S1: Analysis of the cytotoxicity of GS-441524 monotherapy and of the combinatory treatment with fluoxetine.
