# Supplementary material for: Combination Therapy with Fluoxetine and the Nucleoside Analog GS-441524 Exerts Synergistic Antiviral Effects against Different SARS-CoV-2 Variants In Vitro
Source: Pharmaceutics. 2021 Sep 3;13(9):1400. doi: 10.3390/pharmaceutics13091400 (PMC8466181; doi:10.3390/pharmaceutics13091400)
Supplement: Supplementary file 1 [file pharmaceutics-13-01400-s001.zip › pharmaceutics-1327314-supplementary.pdf]

pharmaceutics

Supplementary Material: Combination Therapy with Fluoxetine and the Nucleoside Analog GS-441524 Exerts Synergistic Antiviral Effects against Different SARS-CoV-2 Variants In Vitro

Linda Brunotte, Shuyu Zheng, Angeles Mecate-Zambrano, Jing Tang, Stephan Ludwig, Ursula Rescher and Sebastian Schloer

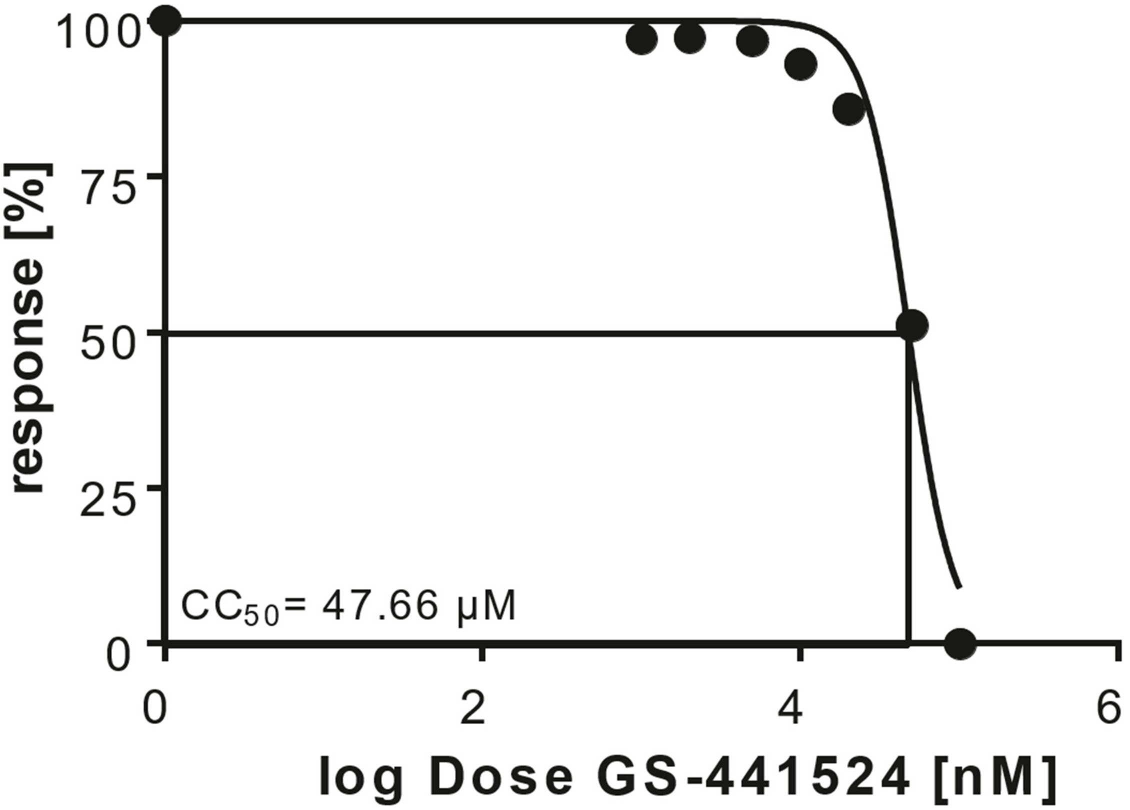

a

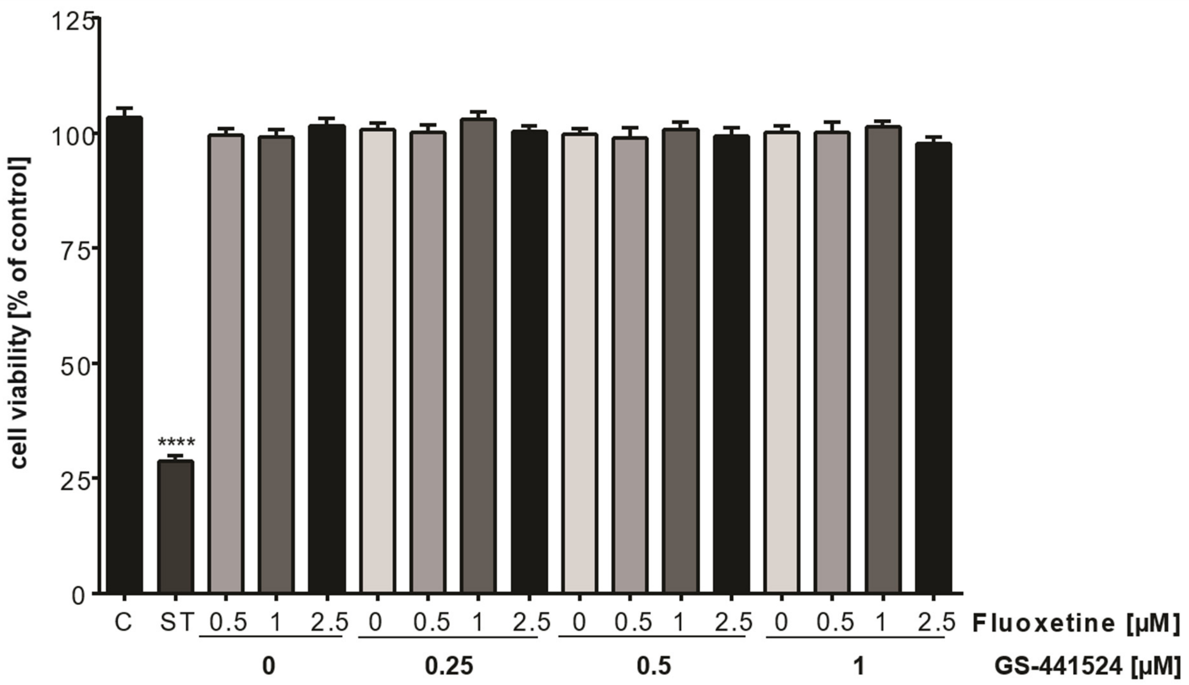

b

Figure S1. Analysis of the cytotoxicity of GS-441524 monotherapy and of the combinatory treatment with fluoxetine. (**a**) GS-441524 single treatment, (**b**) fluoxetine and GS-441524 combinatory treatment. Calu-3 cells were treated with the indicated drug combinations for 48 h. Bars display mean percentages of viable cells ± SEM, with mean viability in solvent-treated control cells (C) set to 100%. Staurosporine (ST)-induced cytotoxicity served as a positive control. n = 5, one-way ANOVA followed by Dunnett's multiple comparison test, ****$p < 0.0001$.
